# Mental health outcomes in patients with cancer diagnosis: Data showing the influence of resilience and coping strategies on post-traumatic growth and post-traumatic symptoms

**DOI:** 10.1016/j.dib.2020.106667

**Published:** 2020-12-24

**Authors:** Alessio Gori, Eleonora Topino, Annamaria Sette, Holger Cramer

**Affiliations:** a Department of Health Sciences, University of Florence, Italy; b Department of Human Sciences, LUMSA University of Rome, Italy; c Department of Internal and Integrative Medicine, Evang, Kliniken Essen-Mitte, Faculty of Medicine, University of Duisburg-Essen, Essen, Germany

**Keywords:** Post-traumatic growth, Impact of event, Coping strategies, Resilience, Oncology, Mental health outcomes, Cancer

## Abstract

The presented data provide a panoramic concerning the influence of different coping strategies in the relationship between resilience and post traumatic growth or post-traumatic symptoms in patients with a cancer diagnosis. These expand the results presented in the related research article of Gori et al. [1], entitled “Pathways to post-traumatic growth in cancer patients: moderated mediation and single mediation analyses with resilience, personality, and coping strategies”. A sample of 154 patients completed a survey, where the Post-Traumatic Growth Inventory, Impact of Event Scale-Revised, Connor-Davidson Resilience Scale, and Coping Orientation to Problems Experienced were included. All the measures for the data collection were in their Italian versions. Data were elaborated by performing single mediation analyses, considering each time the different coping strategies as mediators. The dataset (.xlsx) includes the survey scores, while the tables and figures provide the analysed data, where the mediation models are shown. These data may provide useful bases for future research concerning mental health outcomes in patients with cancer and for tailored programs in preventive or intervention perspectives.

## Specifications Table

**Table:** 

Subject	Psychology (Clinical)
Specific subject area	Mental health outcomes in oncological patients
Type of data	Tables Figures .xlsx file
How data were acquired	Data was gathered using a paper-pencil survey and converted into .xlsx format. Formal analyzes were performed using SPSS software, v.25.
Data format	Raw (upon request) Analyzed
Parameters for data collection	The target population of the survey consisted of individuals diagnosed with cancer, with an age above 18 years.
Description of data collection	A sample of 154 participants completed a paper-pencil survey, after providing written informed consent. They were recruited from various Italian Associations for cancer research and for support of people with cancer, where they filled the questionnaires in approximately 20 min. Privacy and anonymity were guaranteed.
Data source location	Region: Lazio, Tuscany Country: Italy
Data accessibility	The database is not publicly available due to privacy/ethical restrictions. The database linked to this paper can be made available to qualified researchers from the corresponding author upon reasonable request. The Data Use Agreement (DUA) is supplied with the article.
Related research article	A. Gori, E. Topino, A. Sette, H. Cramer, Pathways to post-traumatic growth in cancer patients: moderated mediation and single mediation analyses with resilience, personality, and coping strategies, J. Affect. Disord. 279 (2021) 692–700. https://doi.org/10.1016/j.jad.2020.10.044

## Value of the Data

• The presented data describe significant relationships between resilience, coping strategies and post-traumatic growth or post traumatic symptoms in subjects who received a cancer diagnosis.
• These data highlight different mental health outcomes of patients with cancer and may be useful for researches and professionals working in the oncology sector.
• The data will be useful for researchers who want to conduct comparative studies about the influence of resilience and coping strategies on post-traumatic growth and post-traumatic symptoms in populations with different traumatic life events.
• In the sphere of an application perspective, these data may be useful as a theoretical support in the elaboration of interventions strategies for improving mental health of cancer patients.

## 1 Data Description

The survey includes the Post-Traumatic Growth Inventory ([2] which was administered in its Italian version of Prati and Pietrantoni [3]), Impact of Event Scale-Revised ([4] which was administered in its Italian version of Craparo, Faraci, Rotondo, and Gori [5]), Connor-Davidson Resilience Scale ([6] which was administered in its Italian version of Di Fabio and Palazzeschi [7]), and Coping Orientation to Problems Experienced ([8] which was administered in its Italian version of Sica and colleagues [9]). The dataset (.xlsx file) includes the questionnaires’ total scores of 154 individuals (15 males and 139 females) with a cancer diagnosis and an age ranging from 18 to 79 years (*M* = 51.35; SD = 11.25).

Table 1 describes the models indexes of the mediations of different coping strategies in the relationship between resilience and post-traumatic growth.

**Table 1:** The mediations of different coping strategies in the relationship between resilience and post-traumatic growth: models indexes.

Indirect path: Resilience → mediator → post-traumatic growth							
Mediator	Total effect	Direct effect	Indirect effect	Partially standardized Indirect Effect	Completely Standardized Indirect Effect	Bootstrapping 95% CI	Model Summary
Social Support	1.50	1.36	0.14	0.01	0.04	(−0.021, 0.323)	*R^2^* = 0.321 *F*(2, 150) = 35.427, *p <* .001
Avoidance	1.50	1.60	−0.10	−0.00	−0.03	(−0.223, −0.012)	*R^2^* = 0.262 *F*(2, 150) = 26.594, *p <* .001
Positive Atittude	1.50	0.81	0.69	0.03	0.22	(0.430, 1.037)	*R^2^* = 0.412 *F*(2, 150) = 52.516, *p <* .001
Approach coping	1.50	0.92	0.58	0.03	0.19	(0.329, 0.881)	*R^2^* = 0.363 *F*(2, 150) = 42.806, *p <* .001
Turning to religion	1.50	1.63	−0.13	−0.01	−0.04	(−0.284, −0.025)	*R^2^* = 0.278 *F*(2, 150) = 28.903, *p <* .001

**Fig. 1:**
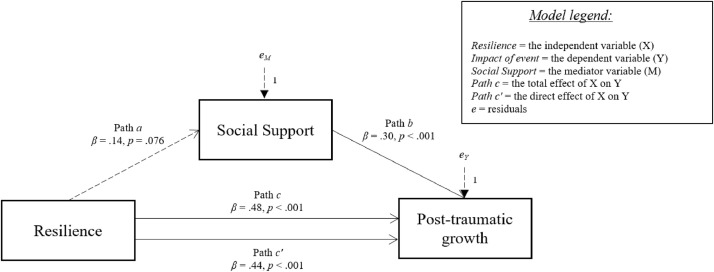
Relationship between resilience and post-traumatic growth with social support coping strategy as mediator.

**Fig. 2:**
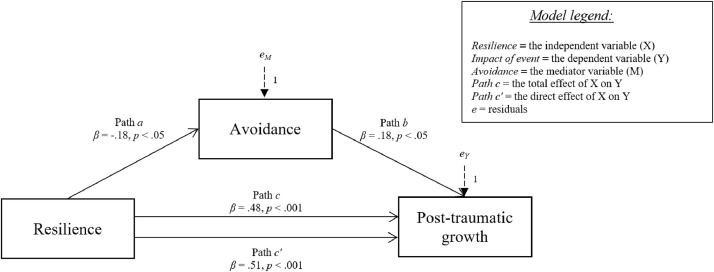
Relationship between resilience and post-traumatic growth with avoidance coping strategy as mediator.

**Fig. 3:**
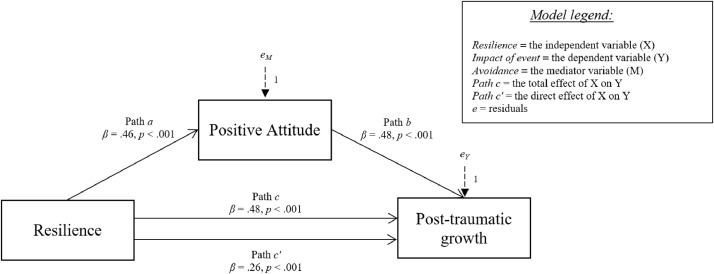
Relationship between resilience and post-traumatic growth with positive attitude coping strategy as mediator.

**Fig. 4:**
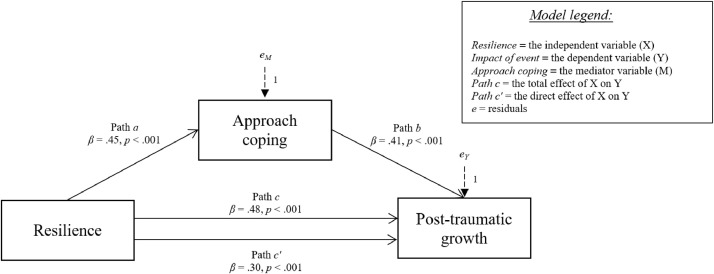
Relationship between resilience and post-traumatic growth with approach coping strategy as mediator.

**Fig. 5:**
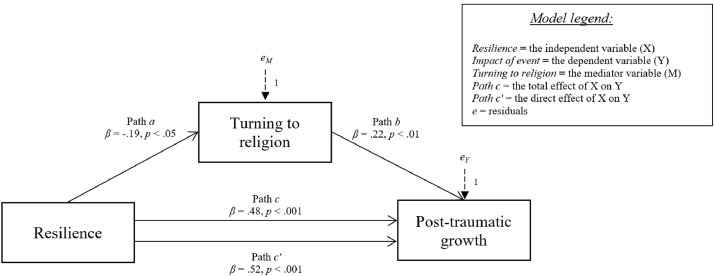
Relationship between resilience and post-traumatic growth with turning to religion coping strategy as mediator.

Fig. 1, Fig. 2, Fig. 3, Fig. 4, Fig. 5 show the mediation models concerning the relationship between resilience and post-traumatic growth.

Table 2 describes the models indexes of the mediations of different coping strategies in the relationship between resilience and impact of trauma.

**Table 2:** The mediations of different coping strategies in the relationship between resilience and post traumatic symptoms: models indexes.

Indirect path: Resilience → mediator → impact of event							
Mediator	Total effect	Direct effect	Indirect effect	Partially standardized Indirect Effect	Completely Standardized Indirect Effect	Bootstrapping 95% CI	Model Summary
Social Support	−0.56	−0.67	0.12	0.01	0.05	(−0.018, 0.302)	*R^2^* = 0.176 *F*(2, 150) = 16.048, *p <* .001
Avoidance	−0.56	−0.43	−0.13	−0.01	−0.06	(−0.255, −0.022)	*R^2^* = 0.146 *F*(2, 150) = 12.772, *p <* .001
Positive Attitude	−0.56	−0.61	0.06	0.00	0.02	(−0.134, 0.262)	*R^2^* = 0.059 *F*(2, 150) = 4.695, *p <* .05
Approach coping	−0.56	−0.85	0.29	0.02	0.13	(0.106, 0.516)	*R^2^* = 0.117 *F*(2, 150) = 9.971, *p <* .001
Turning to religion	−0.56	−0.50	−0.06	−0.00	−0.03	(−0.162, 0.016)	*R^2^* = 0.075 *F*(2, 150) = 6.063, *p <* .01

**Fig. 6:**
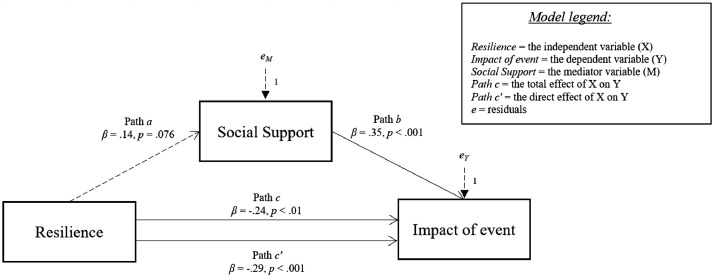
Relationship between resilience and impact of event with social support coping strategy as mediator.

**Fig. 7:**
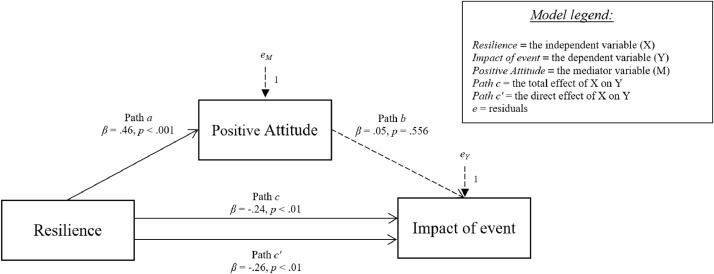
Relationship between resilience and impact of event with positive attitude strategy as mediator.

**Fig. 8:**
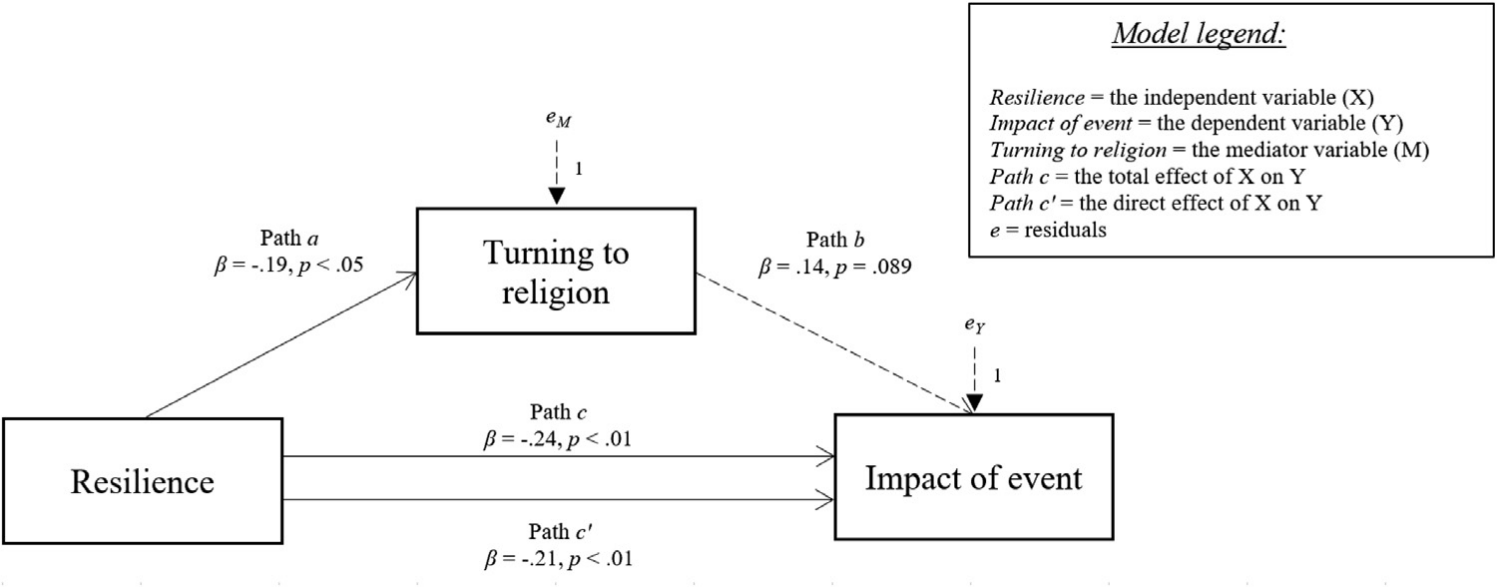
Relationship between resilience and impact of event with turning to religion coping strategy as mediator.

**Fig. 9:**
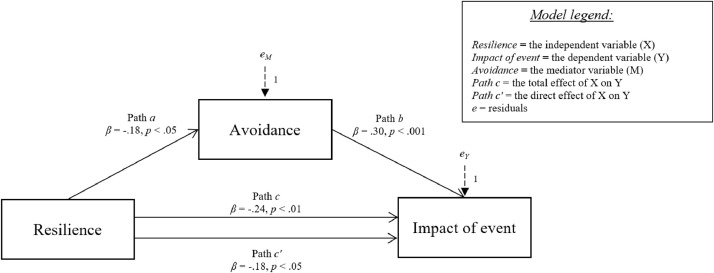
Relationship between resilience and impact of event with avoidance strategy as mediator.

**Fig. 10:**
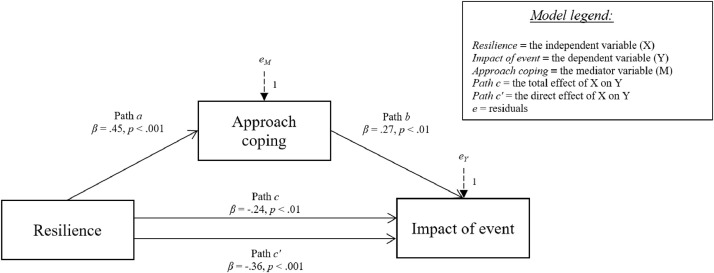
Relationship between resilience and impact of event with approach coping strategy as mediator.

Fig. 6-10 show the mediation models concerning the relationship between resilience and impact of event.

## 2 Experimental Design, Materials and Methods

### 2.1 Participants and procedure

A cross-sectional research design was implemented. A sample of 154 individuals with a cancer diagnosis filled the self-report measures in approximately 20 min. They were recruited in various Italian Associations for cancer research and for the support of people with cancer. Respondents were informed about the aim of the research; their participation was voluntary and anonymous and they signed a written informed consent before starting to complete the survey. The questionnaires were administered by qualified staff authorized to use psychological tests. The research was conducted in accordance with the updated ethical standards and the protocol was approved by the Ethical Committee of the Integrated Psychodynamic Psychotherapy Institute (IPPI) (ethical approval number 002/2019).

### 2.2 Measures

The Italian *Post-Traumatic Growth Inventory* (I-PTGI) [3] was used to assess positive outcomes reported by persons who have experienced traumatic life events [2]. The scale consists of 21 items scored on a 6-point Likert scale from 0 (“*I did not experience this change as a result of my crisis*”) to 5 (“*I experienced this change to a very great degree as a result of my crisis”*). Five subscales were identified: New Possibilities (5 items); Relating to Others (7 items); Personal Strength (4 items); Spiritual Change (2 items); and Appreciation of Life (3 items). In this dataset, the inventory showed good internal consistency, with a Cronbach's alpha ranging from 0.72 to 0.89 for the subscales and α = 0.94 for the total scale.

The Italian *Impact of event scale – revised* (I-IES-R) [5] was used to assess self-reported post-traumatic distress and symptoms [4]. The scale consists of 22 items scored on a 5-point Likert scale ranging from 0 (“*not at all*”) to 4 (“*extremely*”). Three subscales were identified: Intrusion (8 items); Avoidance (8 items); and Hyperarousal (6 items). In this dataset, the measure showed satisfactory internal consistency, with a Cronbach's alpha of 0.89 for the total score and α = 0.67, α = 0.85, α = 0.82 for the subscales.

The Italian *10-item Connor-Davidson Resilience Scale* (I-CD-RISC-10) [7] was used to assess self-perceived resilience [6]. The scale consists of 10 items scored on a 6-point Likert scale ranging from 0 (“*Not true at all*”) to 5 (“*True nearly all the time*”). In this dataset, the measure showed good internal consistency, with a Cronbach's alpha of 0.85.

The *Coping Orientation to Problems Experienced - New Italian Version* (COPE-NIV) [9] was used to assess the respondents’ general coping strategies [8]. The scale consists of 60 items scored on a 4-point Likert ranging from 1 (“*I don't usually do this at all*”) to 4 (“*I usually do this*”). Six factors were identified, all showing satisfactory internal consistency in the present dataset: Social Support (12 items, *α* = 0.82); Avoidance Strategies (16 items, *α* = 0.77); Positive Attitude (12 items, *α* = 0.75); Approach Coping (12 items, *α* = 0.83); and Turning to Religion (8 items, *α* = 0.81).

### 2.3 Statistical analysis

Data entry and analysis were performed using Statistical Package for the Social Sciences (SPSS) software (IBM-SPSS 25.0 version, IBM, Armonk, NY, USA) for Windows. The influences of the coping strategies in the relationships between Resilience and Post-traumatic growth or impact of event were explored by implementing several single mediations using model 4 in macro-program PROCESS 3.4 [10]. The indirect effects were estimated by implementing the bootstrapping technique with 95% CI at 5000 samples.

## Ethics Statement

The authors assert that all procedures contributing to this work comply with the ethical standards. The research protocol was approved by the Ethical Committee of the Integrated Psychodynamic Psychotherapy Institute (IPPI) (ethical approval number 002/2019). Informed consent was obtained from all individual participants included in the study.

## Funding

This research received no specific grants from any funding agency, commercial or not-for-profit sectors.

## CRediT Author Statement

**Alessio Gori**: Conceptualization, Investigation, Formal analysis, Writing - Original Draft, Writing - Review & Editing, Supervision. **Eleonora Topino**: Formal analysis, Writing - Original Draft, Writing - Review & Editing. **Annamaria Sette**: Writing - Review & Editing. **Holger Cramer**: Writing - Original Draft, Writing - Review & Editing, Supervision.

## Declaration of Competing Interest

The authors declare that they have no known competing financial interests or personal relationships which have, or could be perceived to have, influenced the work reported in this article.

## Appendix Supplementary materials

Image, application 1
