# Supplementary material for: Mental health outcomes in patients with cancer diagnosis: Data showing the influence of resilience and coping strategies on post-traumatic growth and post-traumatic symptoms
Source: Data Brief. 2020 Dec 24;34:106667. doi: 10.1016/j.dib.2020.106667 (PMC7773862; doi:10.1016/j.dib.2020.106667)
Supplement: Supplementary file 1 [file mmc1.pdf]

Sample Data Usage Agreement

(Sample DUA for datasets that have de-identified human subject data)

This is an agreement ("Agreement") between you the downloader ("Downloader") and the owner of the materials ("User") governing the use of the materials included in the paper entitled "*Mental health outcomes in patients with cancer diagnosis: data showing the influence of resilience and coping strategies on post-traumatic growth and post-traumatic symptoms*" ("Materials") to be downloaded.

I. Acceptance of this Agreement

By downloading or otherwise accessing the Materials, Downloader represents his/her acceptance of the terms of this Agreement.

II. Modification of this Agreement

Users may modify the terms of this Agreement at any time. However, any modifications to this Agreement will only be effective for downloads subsequent to such modification. No modifications will supersede any previous terms that were in effect at the time of the Downloader's download.

III. Use of the Materials

Use of the Materials include but are not limited to viewing parts or the whole of the content included in the Materials; comparing data or content from the Materials with data or content in other Materials; verifying research results with the content included in the Materials; and extracting and/or appropriating any part of the content included in the Materials for use in other projects, publications, research, or other related work products.

A. Representations

In Use of the Materials, Downloader represents that:

1. Downloader is not bound by any pre-existing legal obligations or other applicable laws that prevent Downloader from downloading or using the Materials;
2. Downloader will not use the Materials in any way prohibited by applicable laws;
3. Downloader has no knowledge of and will therefore not be responsible for any restrictions regarding the use of Materials beyond what is described in this Agreement; and
4. Downloader has no knowledge of and will therefore not be responsible for any inaccuracies and any other such problems with regards to the content of the Materials and the accompanying citation information.

B. Restrictions In his/her Use of the Materials, Downloaders cannot:

1. obtain information from the Materials that results in Downloader or any third party(ies) directly or indirectly identifying any research subjects with the aid of other information acquired elsewhere;
2. produce connections or links among the information included in User's datasets (including information in the Materials), or between the information included in User's datasets (including information in the Materials) and other third-party information that could be used to identify any individuals or organizations, not limited to research subjects; and
3. extract information from the Materials that could aid Downloader in gaining knowledge about or obtaining any means of contacting any subjects already known to Downloader.

IV. Representations and Warranties

USER REPRESENTS THAT USER HAS ALL RIGHTS REQUIRED TO MAKE AVAILABLE AND DISTRIBUTE THE MATERIALS. EXCEPT FOR SUCH REPRESENTATION, THE MATERIALS IS PROVIDED "AS IS" AND "AS AVAILABLE" AND WITHOUT WARRANTY OF ANY KIND, EXPRESS OR IMPLIED, INCLUDING, BUT NOT LIMITED TO, NON-INFRINGEMENT, MERCHANTABILITY AND FITNESS FOR A PARTICULAR PURPOSE, AND ANY WARRANTIES IMPLIED BY ANY COURSE OF PERFORMANCE OR USAGE OF TRADE, ALL OF WHICH ARE EXPRESSLY DISCLAIMED.

WITHOUT LIMITING THE FOREGOING, USER DOES NOT WARRANT THAT: (A) THE MATERIALS ARE ACCURATE, COMPLETE, RELIABLE OR CORRECT; (B) THE MATERIALS FILES WILL BE SECURE ; (C) THE MATERIALS WILL BE AVAILABLE AT ANY PARTICULAR TIME OR LOCATION; (D) ANY DEFECTS OR ERRORS WILL BE CORRECTED; (E) THE MATERIALS AND ACCOMPANYING FILES ARE FREE OF VIRUSES OR OTHER HARMFUL COMPONENTS; OR (F) THE RESULTS OF USING THE MATERIALS WILL MEET DOWNLOADER'S REQUIREMENTS. DOWNLOADER'S USE OF THE MATERIALS IS SOLELY AT DOWNLOADER'S OWN RISK.

V. Limitation of Liability

IN NO EVENT SHALL USER BE LIABLE UNDER CONTRACT, TORT, STRICT LIABILITY, NEGLIGENCE OR ANY OTHER LEGAL THEORY WITH RESPECT TO THE MATERIALS (I) FOR ANY DIRECT DAMAGES, OR (II) FOR ANY LOST PROFITS OR SPECIAL, INDIRECT, INCIDENTAL, PUNITIVE, OR CONSEQUENTIAL DAMAGES OF ANY KIND WHATSOEVER.

VI. Indemnification

Downloader will indemnify and hold User harmless from and against any and all loss, cost, expense, liability, or damage, including, without limitation, all reasonable attorneys' fees and court costs, arising from the i) Downloader's misuse of the Materials; (ii) Downloader's violation of the terms of this Agreement; or (iii) infringement by Downloader or any third party of any intellectual property or other right of any person or entity contained in the Materials. Such losses, costs, expenses, damages, or liabilities shall include, without limitation, all actual, general, special, and consequential damages.

VII. Dispute Resolution

Downloader and User agree that any cause of action arising out of or related to the download or use of the Materials must commence within one (1) year after the cause of action arose; otherwise, such cause of action is permanently barred.

This Agreement shall be governed by and interpreted in accordance with the laws of the Commonwealth of Massachusetts (excluding the conflict of laws rules thereof). All disputes under this Agreement will be resolved in the applicable state or federal courts of Massachusetts. Downloader consents to the jurisdiction of such courts and waives any jurisdictional or venue defenses otherwise available.

VIII. Integration and Severability

This Agreement represents the entire agreement between Downloader and User with respect to the downloading and use of the Materials, and supersedes all prior or contemporaneous communications and proposals (whether oral, written or electronic) between Downloader and User with respect to downloading or using the Materials. If any provision of this Agreement is found to be unenforceable or invalid, that provision will be limited or eliminated to the minimum extent necessary so that the Agreement will otherwise remain in full force and effect and enforceable.

IX. Miscellaneous

User may assign, transfer or delegate any of its rights and obligations hereunder without consent. No agency, partnership, joint venture, or employment relationship is created as a result of the Agreement and neither party has any authority of any kind to bind the other in any respect outside of the terms described within this Agreement. In any action or proceeding to enforce rights under the Agreement, the prevailing party will be entitled to recover costs and attorneys' fees.

Florence, The user

August 04, 2020
